# Electrochemical experiments define potentials associated with binding of substrates and inhibitors to nitrogenase MoFe protein†

**DOI:** 10.1039/d2fd00170e

**Published:** 2023-02-06

**Authors:** Ting Chen, Philip A. Ash, Lance C. Seefeldt, Kylie A. Vincent

**Affiliations:** a Department of Chemistry, University of Oxford, Inorganic Chemistry Laboratory South Parks Road Oxford OX1 3QR UK kylie.vincent@chem.ox.ac.uk; b Department of Chemistry and Biochemistry, Utah State University Logan Utah USA

## Abstract

Nitrogenases catalyse the 6-electron reduction of dinitrogen to ammonia, passing through a series of redox and protonation levels during catalytic substrate reduction. The molybdenum–iron nitrogenase is the most well-studied, but redox potentials associated with proton-coupled transformations between the redox levels of the catalytic MoFe protein have proved difficult to pin down, in part due to a complex electron-transfer pathway from the partner Fe protein, linked to ATP-hydrolysis. Here, we apply electrochemical control to the MoFe protein of *Azotobacter vinelandii* nitrogenase, using europium(iii/ii)-ligand couples as low potential redox mediators. We combine insight from the electrochemical current response with data from gas chromatography and *in situ* infrared spectroscopy, in order to define potentials for the binding of a series of inhibitors (carbon monoxide, methyl isocyanide) to the metallo-catalytic site of the MoFe protein, and the onset of catalytic transformation of alternative substrates (protons and acetylene) by the enzyme. Thus, we associate potentials with the redox levels for inhibition and catalysis by nitrogenase, with relevance to the elusive mechanism of biological nitrogen fixation.

## Abstract 2

We associate potentials with the binding of substrates and inhibitors, H^+^, C_2_H_2_, CO and MeNC, to nitrogenase MoFe protein by coupling electrochemical control with gas chromatography analysis and *in situ* infrared spectroscopy.

## Graphical abstract

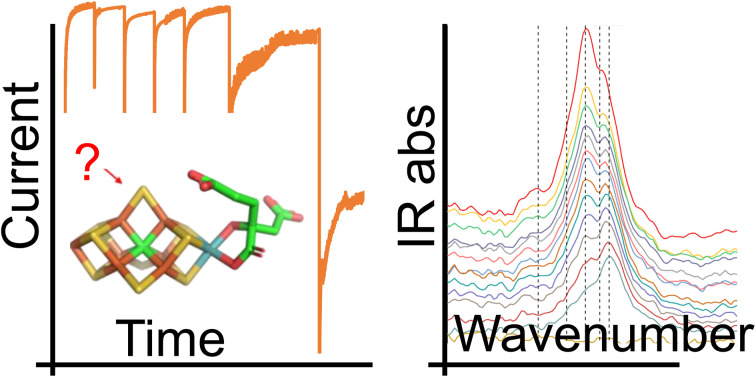

## Introduction

Nitrogenase has held a long fascination for chemists because the enzyme is able to carry out fixation of N_2_ to ammonia in aqueous solution, under ambient conditions. The enzyme has relevance for contemporary efforts to develop electrochemical ammonia synthesis because it essentially operates *via* a redox mechanism, accumulating successive electrons and protons at the catalytic core in the form of metal-bound hydrides, in order to bind and activate the pi-acid ligand, N_2_.^1^ We focus here on molybdenum–iron nitrogenase, rather than the alternative iron–vanadium or iron-only nitrogenases. The catalytic component, the MoFe protein, is a tetramer of polypeptide subunits, comprising two identical subunits denoted α, and two identical subunits denoted β, housing the MoFe_7_S_9_C.*R*-homocitrate catalytic site. Electrons enter the MoFe protein *via* the P cluster, an 8Fe–7S cluster situated at the α–β subunit interface, which undergoes considerable structural change between redox levels.^2,3^ Rounds of electrons are delivered from a partner redox protein known as the Fe protein, which houses a 4Fe–4S redox centre and a site for ATP binding; ATP is hydrolysed to ADP during the electron-transfer cycle. Substantial conformational changes in the protein accompany Fe protein binding, and have been proposed as a gate to electron transfer for the MoFe protein.^4^

Thoughts about the redox levels of the MoFe protein are still informed by a detailed early kinetic model put forward by Lowe and Thorneley.^5^ This proposes a series of states labelled *E*_*n*_, with *n* = 0 to 7 denoting the number of electrons added to the MoFe protein from the *E*_0_ level, with most electron-transfer steps being accompanied by proton transfer. This has been updated in light of more recent mechanistic findings by Hoffman and coworkers,^6^ but spectroscopic and structural detail for the various redox/protonation states of the MoFe protein remains limited.^2^ The indirect nature of electron transfer, *via* the Fe protein, has made it challenging to achieve precise control over the formation of the redox levels of the MoFe protein required for binding of substrates and inhibitors. Variation in the ratio of Fe protein to MoFe protein is often employed to give some control over redox levels of the MoFe protein in kinetic experiments, or in preparation of samples for spectroscopic measurement.^7^

We previously introduced an IR spectroelectrochemical approach to probe proton reduction and CO binding to a genetic variant of nitrogenase MoFe protein in contact with a carbon electrode *via* a series of low-potential redox mediators, in the absence of Fe protein.^8^ These earlier experiments were conducted with the protein trapped in a layer of hydrated Nafion polymer electrolyte. Polymer-entrapped nitrogenase electrodes were also studied electrochemically by Milton *et al.*, for reduction of N_3_^−^ and NO_2_^−^ to ammonia with cobaltocenium/cobaltocene as a redox mediator.^9^ In the present study, we apply an electrochemical and spectroelectrochemical approach in which the nitrogenase MoFe protein is directly immobilised on the electrode surface, overcoming several disadvantages of our earlier spectroelectrochemical approach – in particular, strong IR absorption from the Nafion below 1400 cm^−1^, and impeded access of small molecule solutes through the Nafion layer, and slow leaching of protein from the Nafion layer. Here, the MoFe protein is attached to the carbon electrode surface, either by direct adsorption or by covalent attachment using a carbodiimide coupling protocol, linking carboxylic acid groups introduced onto the carbon surface *via* adsorption of pyrene carboxylic acid, with surface lysines on the protein.^10^ A series of mediators which exploit the Eu(iii)/(ii) couple of europium polyaminocarboxylate complexes are employed to assist electron transfer from the electrode to the protein in a negative potential regime (see ESI, Scheme S1 and Fig. S1 and S2†).^11^

For the nitrogenase MoFe protein adsorbed on a carbon electrode surface, we examine electrocatalytic proton reduction, and explore the potential-dependent interactions of carbon monoxide (CO), acetylene (C_2_H_2_) and methyl isocyanide (MeNC) with the protein. Proton reduction is an important side reaction of MoFe nitrogenase, and occurs in the absence of other substrates as well as accompanying N_2_ reduction. Acetylene has long been known as an effective, non-natural substrate of MoFe nitrogenase, reduced to ethylene (C_2_H_4_) under typical biochemical assay conditions with the Fe protein/ATP as reductant.^7^ The pi-acid ligand, methyl isocyanide, is reduced to a mixture of methane, methylamine and dimethylamine by the nitrogenase MoFe protein in biochemical assays using Fe protein as the reductant, with a small amount of ethylene and ethane also formed.^7^ Methyl isocyanide is thought to act as both inhibitor and substrate for the MoFe protein, and can completely suppress proton reduction. Reduction of MeNC occurs at a low ratio of Fe protein to MoFe protein, suggestive of binding at a less reduced level than that required for reduction of N_2_ or C_2_H_2_.^6^

Carbon monoxide, also a pi-acceptor ligand, and isoelectronic with N_2_ and isocyanides, is a well-established inhibitor of the reduction of most substrates of MoFe nitrogenase. Proton reduction by MoFe nitrogenase is not inhibited by CO in biochemical assays,^12,13^ in contrast to the iron and vanadium nitrogenases.^13^ The binding of CO to MoFe nitrogenase is complex, and a number of different binding modes are intimated by stopped-flow IR studies at different CO concentrations and by EPR/ENDOR studies.^4,14^ A protein crystallographic structure with a single CO ligand bound to the MoFe protein of nitrogenase was achieved by crystallisation of enzyme under turnover conditions in the presence of CO, and reveals a bridged binding mode between two Fe atoms in the ‘waist’ region of the FeMo cofactor, with CO replacing one of the waist sulfur atoms.^15^ Preparation of crystals under *ca.* 5.5 bar CO resulted in a structure with two CO ligands bound, with the second CO coordinated in a terminal binding mode to one of the same ‘waist’ Fe atoms, at the Mo end of the cluster.^16^ Binding of two CO ligands in close proximity is likely to account for the slow C–C forming reduction of CO to hydrocarbons by the nitrogenases.^13,17,18^

The ability to observe ligand binding and substrate reduction by the MoFe protein, in the absence of Fe protein and ATP, enables us to associate potentials with these events, in a way that has been difficult to achieve through conventional biochemical assays.

## Materials and methods

### Proteins and reagents

Native nitrogenase MoFe protein from *Azotobacter* (*A.*) *vinelandii* was prepared as described previously.^19^ The apo protein, lacking the MoFe catalytic site,^20^ was also used for control experiments. Experiments were performed inside an anaerobic glove box (Glove Box Technology, <2 ppm O_2_). Gases CO and Ar were purified by passing through an O_2_ scrubbing filter (Restek Super Clean gas filter, <1 ppm O_2_) before bubbling into solutions inside the anaerobic glove box, with flow rates controlled using a mass flow controller (Brooks Instruments). For electrochemical and IR spectroelectrochemical experiments, acetylene was generated by reacting calcium carbide with deoxygenated MilliQ water in a gas bottle contained within the anaerobic glovebox (see ESI, Fig. S3†), whereas for gas chromatographic experiments, acetylene was provided from a cylinder.

Electrochemical experiments were conducted in 100 mM Tris–HCl buffer, pH 8.0, containing 100 mM NaCl, prepared in MilliQ water (resistivity, 18 mΩ cm). A cocktail of redox mediators was added to the electrolyte, comprising three europium(iii/ii) ligand complexes (collectively termed ‘Eu-L’ hereafter), where the ligands are: 1,2-bis(*o*-aminophenoxy)ethane-*N*,*N*,*N′*,*N′*-tetraacetate (BAPTA), ethylene glycol-bis(β-aminoethyl ether)-*N*,*N*,*N′*,*N′*-tetraacetate (EGTA), and diethylenetriamine-*N*,*N*,*N′*,*N′′*,*N′′*-pentaacetate (DTPA) (see ESI, Scheme S1†). These have been used previously in studies of nitrogenase.^8^ Potentials were determined in the experimental buffer solution by cyclic voltammetry (ESI, Fig. S1†), giving potentials (*vs.* the standard hydrogen electrode, SHE) consistent with previous studies: Eu-BAPTA, −0.63 V; Eu-EGTA, −0.865 V; and Eu-DTPA, −1.087 V. The cobaltocene/cobaltocenium couple, which has a potential similar to Eu(iii/ii)-EGTA, has been used previously as a mediator for MoFe nitrogenase,^9^ but was found to be unsuitable here due to strong precipitation or adsorption onto the electrode surface.

Methyl isocyanide was prepared as previously reported.^21^ Isocyanide solutions were prepared by injecting neat isocyanide into the deoxygenated Eu-L mediator solution in a sealed crimp-top vial in the fume hood and moving immediately to an anaerobic glovebox.

### Preparation of nitrogenase electrodes

Nitrogenase MoFe protein was immobilised onto high surface area carbon black particles (BP2000, Cabot) *via* direct adsorption of 2 nmol protein onto 5 μL of a 20 mg mL^−1^ suspension of BP2000, on ice, for 2 hours. Alternatively, the protein was covalently attached onto BP2000: the particles were first modified with pyrene carboxylic acid which pi-stacks onto the carbon surface; an aliquot of 20 mg mL^−1^ BP2000 was mixed with 10 mM 1-pyrenebutyric acid (Sigma) in dimethyl formamide (DMF) and stored at 4 °C for 10 hours; the suspension was then rinsed with DMF and water to remove any excess pyrenebutyric acid. The free carboxylic acid group was then activated for 1 hour using a freshly prepared solution of 0.4 M 1-ethyl-3-(3-dimethylaminopropyl) carbodiimide (EDC) and 0.1 M *N*-hydroxy sulfosuccinimide (NHSS) in the glovebox, before treating with 2 nmol nitrogenase MoFe protein for 4 hours, to make covalent attachments *via* exposed lysine residues on the protein surface. Non-coupled protein was then rinsed off with buffer. Particles modified with MoFe protein by either direct adsorption or covalent attachment were then coated onto the electrochemical cell baseplate or silicon ATR-IR internal reflection element. Monitoring of the amide II band at *ca.* 1550 cm^−1^ showed poor stability over a 5 hours spectroscopic experiment for the directly adsorbed protein, but excellent stability for the covalently attached protein (see ESI, Fig. S2†).

### Electrochemistry and IR spectroelectrochemistry

Electrochemical control was imposed by a potentiostat (AutoLab or PalmSens). Infrared spectroelectrochemical experiments were performed on a Varian 680-IR spectrometer equipped with a GladiATR™ accessory (PIKE Technology, custom-modified) and liquid N_2_ cooled MCT detector, housed inside an anaerobic glovebox, as described previously.^8^ Electrochemical and IR spectroelectrochemical measurements were performed in a custom-built sample cell which has also been described previously.^8^ In brief, the carbon particle working electrode is coated onto a multi-bounce silicon internal reflection element to allow attenuated total reflectance detection of the electrode–electrolyte layer. A Pt wire counter electrode and home-built miniature saturated calomel reference electrode are positioned in a solution compartment above the working electrode. Connection to the working electrode layer was made by a carbon rod. Electrolyte solution was continuously circulated through the cell during measurements using a peristaltic pump (Watson Marlow 120U) with 0.1 mm diameter tubing, and the electrolyte solution was gas-saturated in a separate solution reservoir *via* syringe needles through a septum-sealed cap (see ESI, Fig. S3†). Spectra were recorded as 512 co-added scans, with a resolution of 4 cm^−1^.

### Gas chromatographic detection of ethylene product

A PalmSens 4 potentiostat provided the electrochemical control for experiments coupled with GC headspace sampling. The electrochemical cell was identical to that used for the electrochemical and IR spectroelectrochemical measurements. Concentrations of C_2_H_4_ were calculated from GC data *via* comparison to a standard curve prepared with an ethylene gas standard.^18,22^

## Results

### Electrocatalytic proton reduction and CO inhibition for carbon-immobilised nitrogenase MoFe protein

**Fig. 1:**
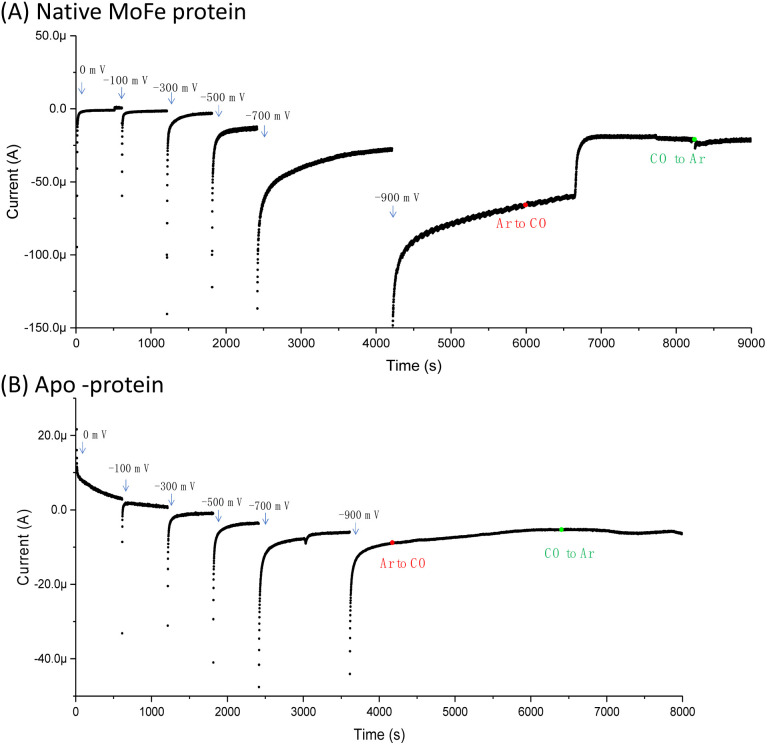
Comparison of the current response for an electrode modified with (A) native MoFe protein or (B) apo-protein. In the first part of the experiment, buffer saturated with argon (Ar) was flowed through the cell from a gas-saturated reservoir *via* a peristaltic pump. At the point shown by the red dot, the gas flow into the buffer reservoir was changed from Ar to carbon monoxide (CO); a lag phase of *ca.* 650 s results before this solution reaches the electrochemical cell due to the length of the pump tubing. At the point marked by a green dot, the gas flow in the buffer reservoir was switched back to Ar, and again there is a lag phase before this reaches the electrochemical cell. In each case, the buffer was Tris HCl, pH 8.0, containing Eu-L mediators.

Fig. 1A shows the electrochemical response for nitrogenase MoFe protein on a carbon electrode in Ar-saturated buffer at different potentials. The mild increase in the magnitude of the negative current at each successive potential step as far as −0.5 V is consistent with capacitive (non-faradaic) current and is similar to the response observed when the electrode is instead modified with apo-MoFe protein, lacking the active site FeMo-cofactor (Fig. 1B). Upon stepping to −0.7 V, the current shows a more significant increase in magnitude for native MoFe protein, and increases further at −0.9 V, consistent with electrocatalytic proton reduction by the enzyme, which was also observed in experiments conducted on the same protein trapped in a Nafion electrolyte film.^8^ With the electrode poised at −0.9 V, the gas flushing into a buffer reservoir was changed from Ar to CO; there is a lag phase of *ca.* 650 s before CO-saturated buffer reaches the electrochemical cell *via* peristaltic pump and tubing, but at *ca.* 750 s, the current is rapidly attenuated, almost to zero, consistent with CO inhibition of electroenzymatic proton reduction. No recovery of current was observed when the gas flushing into the buffer reservoir was exchanged back to Ar, showing that this inhibition is irreversible at −0.9 V. In contrast, exchanging CO for Ar has a negligible effect on the current for the apo-protein, supporting interpretation of the result in Fig. 1A as CO binding at the FeMo-cofactor active site and affecting electrocatalysis. When the whole potential sequence was repeated with CO-saturated buffer solution in the cell from the beginning of the experiment (ESI, Fig. S4A†), there was no significant increase in current observed at −0.7 or −0.9 V, consistent with proton reduction being inhibited. Another potential step experiment conducted under a N_2_ atmosphere (ESI Fig. S4B†) showed similar current responses to the Ar experiment shown in Fig. 1A, within the variation observed from one protein film to another, hence providing no evidence for electrocatalytic N_2_ reduction and no evidence for N_2_ affecting electrocatalytic proton reduction.

**Fig. 2:**
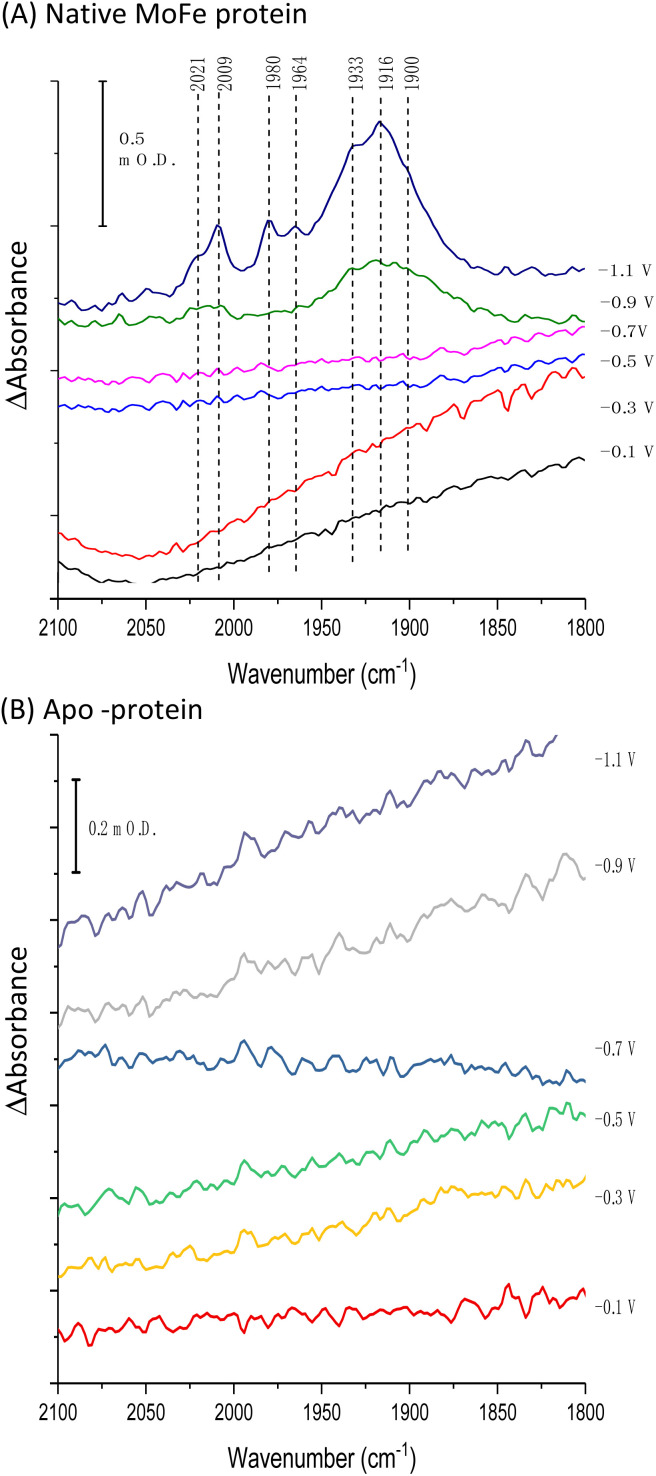
IR spectra, showing the *v*_CO_ region, recorded *in situ* during electrochemical control of a film of carbon black particles modified with (A) native MoFe protein or (B) apo-protein. Spectra were recorded in CO-saturated Tris HCl, pH 8.0 electrolyte containing Eu-L. After each potential step, the electrode was poised for at least 30 minutes, or until no further spectral changes were observed, before the presented IR spectra were recorded. All spectra are processed against a background scan recorded at −0.1 V before introduction of CO to the cell.

Fig. 2A shows IR spectra recorded during a potential step sequence on adsorbed MoFe protein, with CO-saturated buffer in the cell. The region of the IR spectrum characteristic for the CO-stretching (*v*_CO_) bands of metal-bound CO ligands is shown. Changes to the baseline slope arise because this region of the spectrum overlaps the broad combination band of water, which is likely affected by potential-dependent rearrangement of water near to the electrode surface (see ESI, Fig. S5†). No evidence for coordination of CO is observed in the spectra recorded at −0.1, −0.3, −0.5 or −0.7 V, but a broad peak centred at around 1920 cm^−1^ appears at −0.9 V, together with a weaker feature above 2000 cm^−1^. These features sharpen at −1.1 V to reveal a complex mixture of bands consistent with multiple modes of CO binding or CO bound at multiple redox levels. Assignment of these peaks to CO binding to the FeMo-cofactor of MoFe-nitrogenase is supported by an analogous experiment on the apo-protein (Fig. 2B), in which no *v*_CO_ bands are observed at any potential. The role of the Eu-L mediators in providing effective electron transfer to the electrode-adsorbed MoFe protein is shown by an analogous experiment performed in the absence of mediators, ESI Fig. S6,† in which no spectral bands attributable to *v*_CO_ are observed after a 60 minutes poise at −0.9 V, and only a very minor feature is observed around 2000 cm^−1^ after a 60 minutes poise at −1.1 V. This may be because some molecules of MoFe protein are incorrectly orientated for direct electron transfer.

**Fig. 3:**
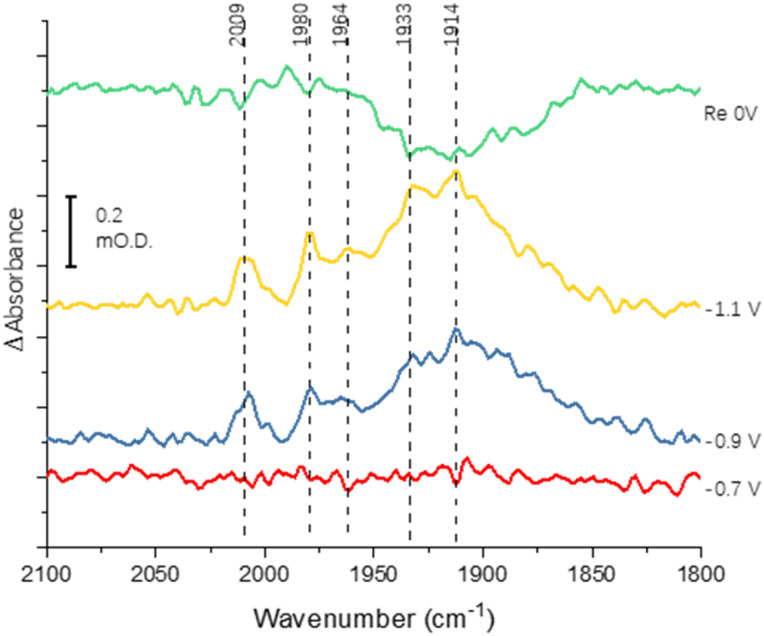
Spectra recorded to probe reversibility of CO binding to native MoFe protein. The protein was covalently attached to the electrode using the carbodiimide coupling reaction. Spectra were recorded at −0.7, −0.9 and −1.1 V relative to a background scan recorded at 0 V. The spectrum labelled ‘Re 0 V’ was recorded following a subsequent step back to 0 V, *vs.* a background of the previous scan (−1.1 V).

Fig. 3 shows the results of an experiment designed to probe reversibility of CO binding. To allow for the longer experiment, the protein was covalently immobilised onto the electrode *via* the carbodiimide coupling step described in the Materials and methods section. Spectra at −0.7, −0.9 and −1.1 V are qualitatively comparable to those shown for the electrode-adsorbed film in Fig. 2. The top spectrum shows the effect of re-oxidation at 0 V, and is recorded *vs.* a background at −1.1 V. Although there is no clear depletion of the sharper peaks at 2009, 1980 and 1964 cm^−1^, the broad peak centred around 1920 cm^−1^ shows significant depletion after the re-oxidation step, indicating significant reversibility of CO binding. In future work it will be productive to explore whether proton reduction at low potentials is restored after flushing out CO with Ar at 0 V.

**Fig. 4:**
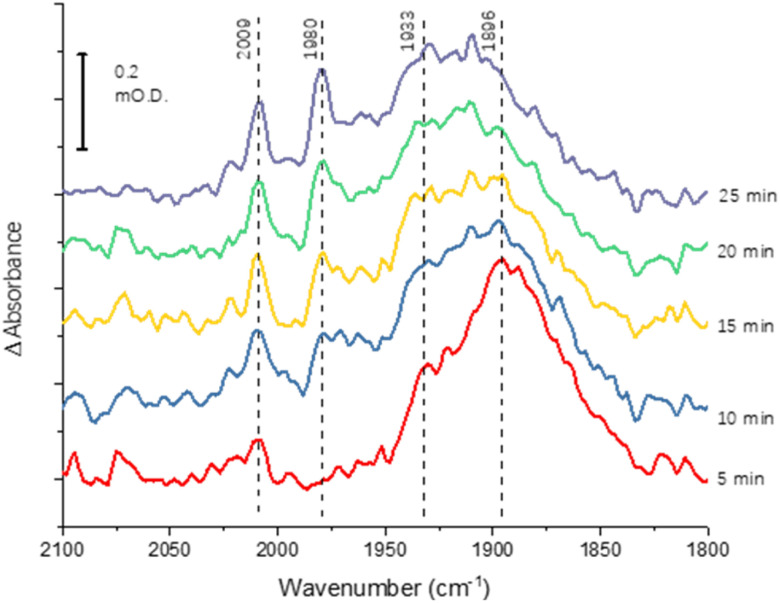
Time-course spectra for CO binding to nitrogenase MoFe protein at −0.9 V. The protein was covalently attached to the electrode using the carbodiimide coupling reaction. Spectra are calculated from a background at −0.7 V, recorded prior to the step to −0.9 V.

The greater coverage and stability of the covalently attached protein enabled us to record a time-course following introduction of CO to the cell, Fig. 4. Some temporal dependence of the *v*_CO_ bands of coordinated CO is evident, with a low wavenumber feature below 1900 cm^−1^ emerging first, possibly due to initial coordination of CO in a bridging mode. Subsequently, the shoulder at around 1930 cm^−1^ increases in intensity, accompanied by appearance of higher wavenumber peaks at 2009 and 1980 cm^−1^, which would be consistent with a shift from bridging to terminal CO coordination, as suggested in a range of earlier studies of the MoFe protein reduced by Fe protein/ATP in the presence of CO.^14–16,23^

### Acetylene reduction

**Fig. 5:**
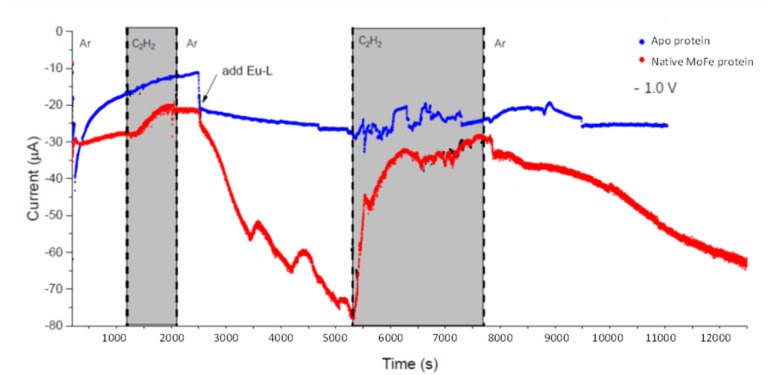
Electrochemical experiment at −1.0 V examining the effect of C_2_H_2_ on electrocatalytic proton reduction by nitrogenase MoFe protein and apo protein. At the commencement of the experiment, the buffer flowing through the cell contained no Eu-L mixture, but Eu-L was introduced at 2500 s. The buffer in the solution reservoir was flushed with Ar, except in the periods marked in grey, when C_2_H_2_ was flushed through the solution reservoir.

We next conducted experiments in which acetylene (C_2_H_2_) was introduced during electrochemical control of an electrode modified with native MoFe protein, again using the apo protein electrode for comparison. In the experiment shown in Fig. 5, the cell was first flushed with Tris HCl pH 8.0 buffer saturated with Ar, with no Eu-L mediators. The modest negative current observed for the native protein, in contrast to the apo-protein, under these conditions suggests that there must be some direct electron transfer from the electrode to the native enzyme to enable unmediated electrocatalytic proton reduction by the enzyme. This current is attenuated when C_2_H_2_ is introduced; this could be due to inhibition of H^+^ reduction by C_2_H_2_, or to competing, slower C_2_H_2_ reduction by the enzyme, giving an overall lower current magnitude. The apo protein electrode shows no effect of C_2_H_2_, supporting assignment of the effect of C_2_H_2_ on native MoFe protein as occurring at the active site.

At 2500 s, the Eu-L mixture was injected into the solution reservoir, after re-equilibrating with Ar. This gave rise to a slow but significant increase in current magnitude as the mediators equilibrated with the cell solution, showing that the mediated electron transfer to nitrogenase is substantially more effective than the direct electron transfer at carbon. At about 5300 s, C_2_H_2_ was re-introduced, and, for native nitrogenase MoFe protein, again resulted in a dramatic attenuation of the proton reduction current. The effect is partially reversible: a slow restoration of current is observed after returning to Ar flushing through the solution reservoir. (Instability in the current in the presence of C_2_H_2_ results from fluctuations in pressure in response to addition of solid CaC_2_ to the gas bottle.)

**Fig. 6:**
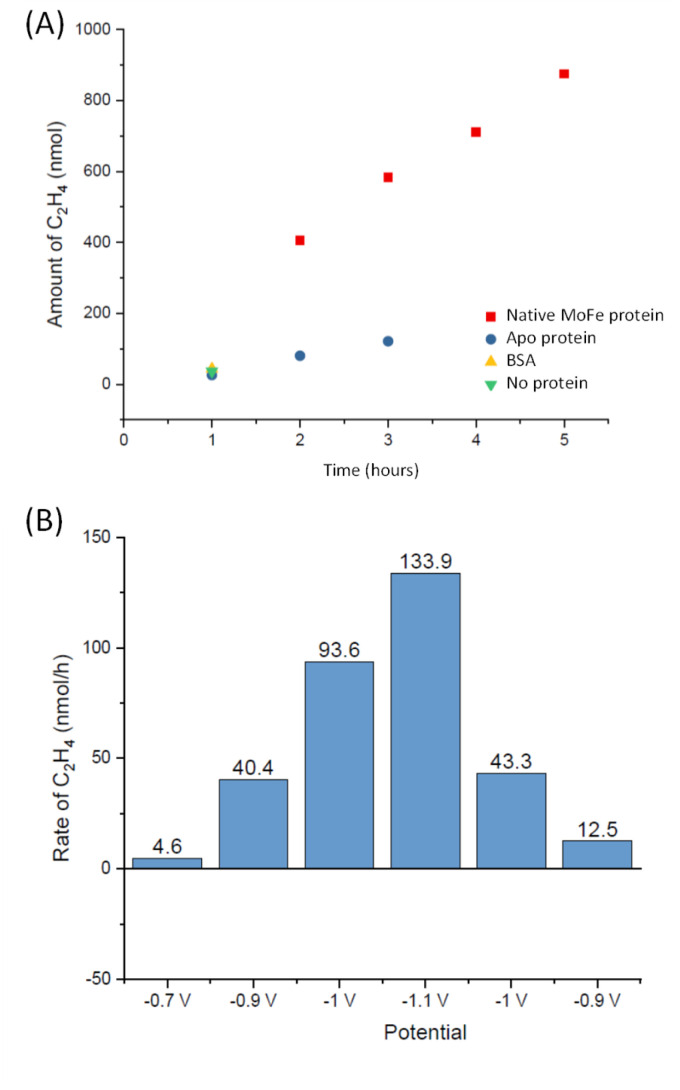
Experiments to quantify C_2_H_4_ production at MoFe protein electrodes. (A) Calculated headspace yield of C_2_H_4_ (GC) from experiments at −1.1 V for electrode modified with native MoFe protein (red squares) or apo protein (blue circles) over time. The value at 1 hour is also shown for an electrode similarly modified with bovine serum albumin (BSA, yellow triangle), or simply activated with EDC and NHSS (green triangle). (B) Calculated headspace yield of C_2_H_4_ for a separate electrode-preparation of native MoFe protein, subjected to different potential steps.

To explore whether the current attenuation correlated with suppression of H^+^ reduction by slower electrocatalytic C_2_H_2_ reduction to ethylene, C_2_H_4_, Eu-L mediated electrochemical experiments were coupled with gas chromatography (GC) sampling of the headspace to assess the presence of ethylene during a reaction in which acetylene from a cylinder was flushed into the solution reservoir. Fig. 6A shows the results of C_2_H_4_ determination over time for native MoFe protein and apo protein. Some background C_2_H_4_ production is observed with the electrode modified with apo protein, giving a rate of 2.5 nmol min^−1^ (mg protein)^−1^ at 3 hours. However, the C_2_H_4_ production from native MoFe protein is significantly higher, giving a rate of 12.6 nmol min^−1^ (mg protein)^−1^ at 3 hours, and representing a faradaic efficiency for the 2-electron reduction of C_2_H_2_ to C_2_H_4_ by native nitrogenase MoFe protein of *ca.* 28%. An overall rate of *ca.* 10 nmol min^−1^ (mg protein)^−1^ is maintained over 5 hours. Further control experiments in which the electrode was modified with a non-catalytic protein, bovine serum albumin (BSA), or simply activated with EDC and NHSS (see Materials and methods section), were performed over a shorter timeframe of 1 hour, and gave low background C_2_H_4_ levels similar to the apo-protein experiment.

The experiment shown in Fig. 6B was performed on a separate electrode preparation of native MoFe protein and shows the C_2_H_4_ yield after 1 hour of reaction at each potential indicated. In this experiment, the poise at −1.1 V gives a C_2_H_4_ production rate of 8.5 nmol min^−1^ (mg protein)^−1^, still significantly above the apo-protein background noted above. Variation in overall activity between different electrode preparations is expected due to differences in the coverage of protein or the quantity of carbon particles addressed electrochemically, and likely loss of some activity during the longer potential step sequence used here, but this gives an indication of qualitative reproducibility of the system. Importantly, this experiment indicates an onset potential for C_2_H_2_ reduction of around −0.9 V, similar to the onset for CO binding and electrocatalytic proton reduction.

### MeNC interaction

**Fig. 7:**
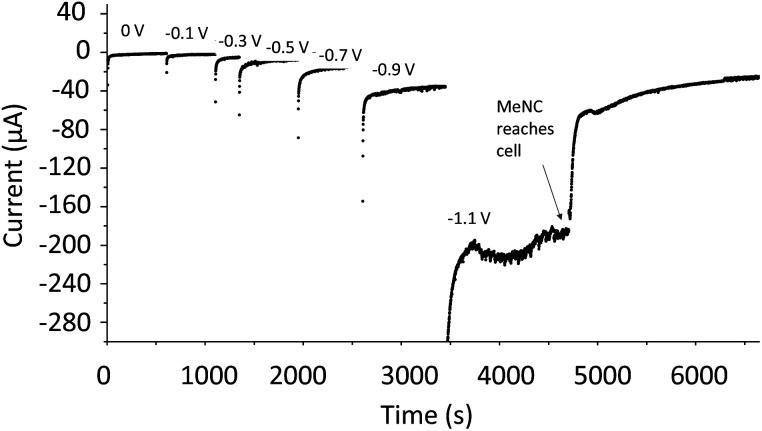
Potential step sequence demonstrating attenuation of H^+^ reduction current by native MoFe protein at −1.1 V when MeNC reaches the electrochemical cell (25 mM).

**Fig. 8:**
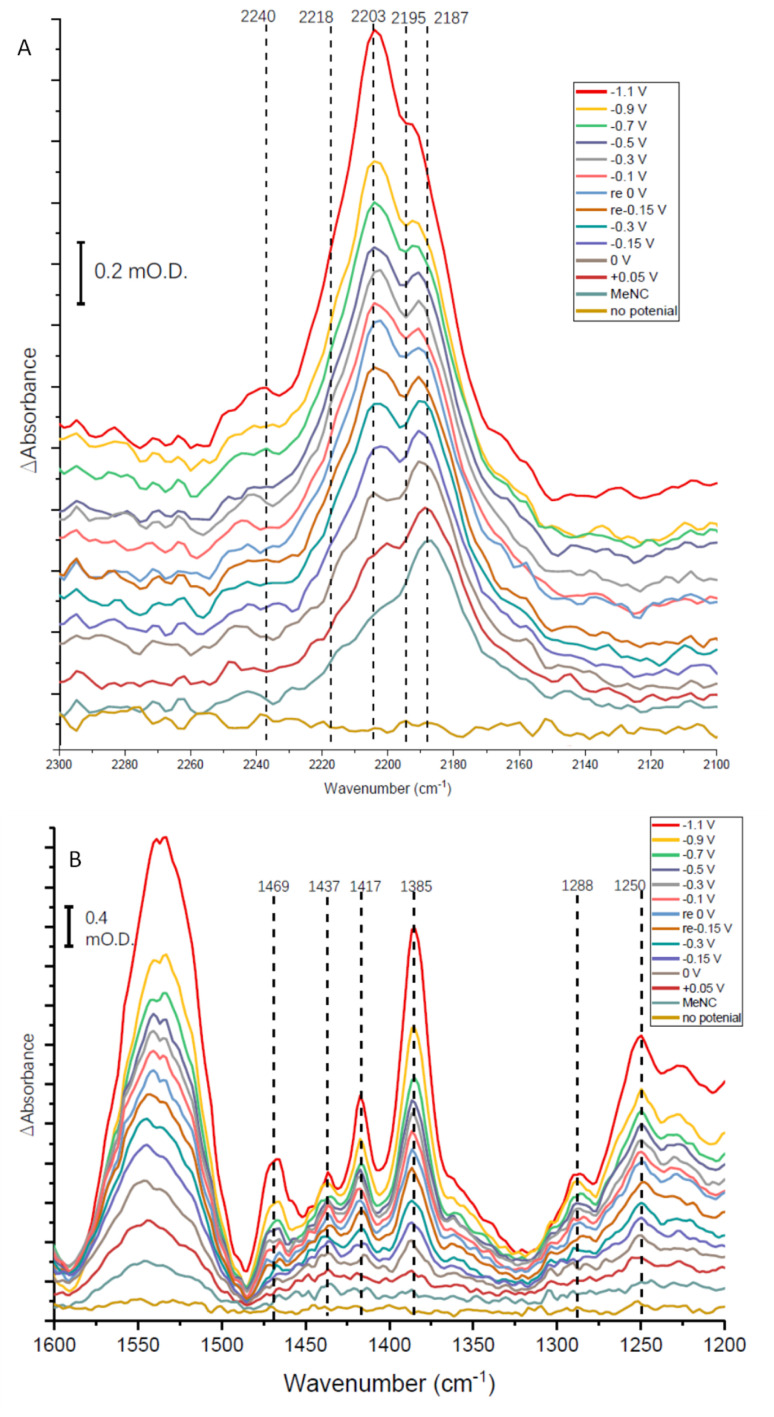
IR spectra recorded over a series of potentials for an electrode modified with native nitrogenase MoFe protein in Tris HCl pH 8.0 buffer in the presence of Eu-L mediators for 25 mM methyl isocyanide, MeNC. Potentials were applied in the order indicated (bottom to top); potentials marked ‘re’ in the legend indicate a re-oxidation stage after the application of the first mild reducing potentials, before application of more negative potentials again; ‘no potential’ indicates open circuit conditions. Panels A and B show different parts of the mid-IR spectrum. Spectra were each recorded 30 minutes after applying the potential indicated, and are each processed against a spectrum recorded at open circuit potential (no applied potential) before introduction of MeNC.

Methyl isocyanide is known as both an inhibitor and an alternative substrate of MoFe nitrogenase.^7^ We first analysed the effect of MeNC on electrocatalytic proton reduction at low potentials where we have observed CO to interact. Attenuation of the current following introduction of MeNC indicates inhibition of proton reduction (Fig. 7). We then investigated regions of the IR spectrum where peaks from the *v*_NC_ stretch of free or coordinated MeNC would be expected (Fig. 8A), as well as a lower wavenumber region (Fig. 8B) where NH and CH deformation vibrations of the reduced product, methylamine, might be expected.

The absorption spectra shown in Fig. 8A show a broad absorption feature in the *v*_NC_ region after addition of MeNC, even when no potential is applied. The *v*_NC_ stretch of free MeNC dissolved in aqueous solution occurs at 2187 cm^−1^. Free MeNC is seen in the first spectrum after addition of MeNC, with no potential applied, but additional higher wavenumber peaks are also evident under the broad absorption feature. These resolve into sharper peaks around 2203 and 2195 cm^−1^, with a higher wavenumber shoulder around 2218 cm^−1^, as the potential is stepped more negative, with a further high wavenumber peak at around 2240 cm^−1^ appearing at more negative potentials. Notably, when the apo protein is subjected to a potential of −1.1 V in the presence of MeNC, only the 2187 cm^−1^ peak of free MeNC is observed (ESI Fig. S7†). The contrast between native MoFe protein and apo protein spectra with MeNC suggests that the native MoFe protein coordinates MeNC, even at around 0 V.

Several peaks are observed to grow in the low wavenumber range (Fig. 8B). In particular, a shoulder 1520 cm^−1^ and clear peak at 1469 cm^−1^ would be consistent with the strongest absorption peaks of methylamine in aqueous solution (see ESI, Fig. S8†), and are completely absent in spectra recorded for apo-protein under identical experimental conditions (ESI, Fig. S8†), suggesting catalytic reduction of MeNC to methylamine by MoFe protein. The 1469 cm^−1^ peak grows in with increasingly negative potential, and is first evident at around −0.1 V (Fig. 8B), suggesting MeNC reduction commences at a very mild potential. No redox mediators suitable for controlling the potential above *ca.* −0.5 V were included, so potential control in this region relies on direct electron transfer to the protein from the electrode.

In a separate experiment, the IR absorption for free MeNC at 2187 cm^−1^ was monitored over 15 hours with the potential poised at −1.1 V and a stationary solution (*i.e.* not pumped through the cell). During this time, *ca.* 65% of the intensity at 2187 cm^−1^ was depleted, consistent with consumption of the majority of the MeNC in the cell solution (ESI, Fig. S9†). In contrast, the intensity loss when the experiment was repeated with a film of apo protein was less than 10%, lending additional weight to the suggestion that the MoFe protein catalyses MeNC electroreduction.

## Discussion

**Fig. 9:**
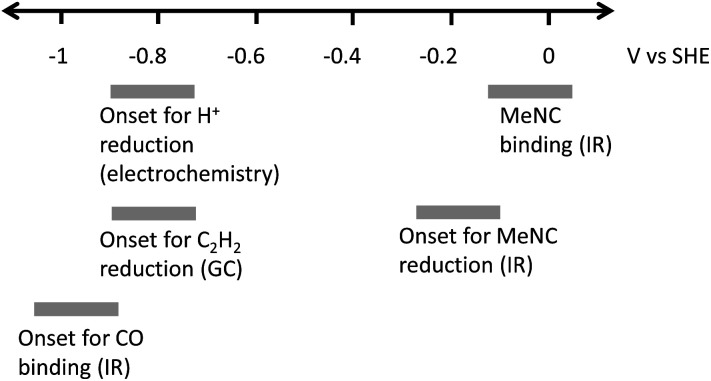
Potentials observed for binding or reduction of different ligands to nitrogenase MoFe protein immobilised on a carbon electrode at pH 8.0. The means of detecting the interaction are indicated in parentheses. The grey bars represent potential ranges due to the 200 mV potential steps applied in most of the experiments described in this study.

Studies of the binding of different ligands to nitrogenase MoFe protein, and the catalytic reduction of different substrates, are typically controlled indirectly by modulating electron flux *via* the ratio of electron-transfer Fe proteins to catalytic MoFe protein.^4,7^ The indirect nature of this electronic control has made it difficult to associate particular redox levels with ligand binding events. Here, we have employed an electrochemical methodology, coupled with *in situ* infrared spectroscopy or external GC headspace gas sampling, to associate the binding of ligands and catalytic reduction of several substrates with particular potential ranges, Fig. 9. By use of a series of Eu-L complexes as mediators spanning the negative potential range, we achieve control over the redox chemistry of the MoFe protein in the absence of Fe protein and ATP. Covalent attachment of the MoFe protein onto the carbon electrode surface provides enzyme films which are sufficiently robust for these studies to be carried out over a number of hours.

Proton reduction is observed *via* the negative electrocatalytic current, with an onset in the range −0.7 to −0.9 V *vs.* SHE, in contrast to the low background current in this range from the apo protein (which lacks the FeMo-cofactor catalytic site), studied under identical conditions (Fig. 1). Further confirmation for this being electroenzymatic activity arising from the MoFe protein is given by the CO inhibition step in Fig. 1, which shows that the current at −0.9 V for the native MoFe protein electrode, but not the apo-protein electrode, depletes significantly on exposure to CO, apparently back to the background level. Proton reduction by MoFe protein in biochemical assays with Fe protein/ATP as reductant has been reported to be insensitive to CO inhibition,^7,13^ although interestingly, the alternative VFe and Fe nitrogenase do show susceptibility of H^+^ reduction to inhibition by CO, as well as CO reduction to hydrocarbons.^13^ The significant inhibition of electrocatalytic proton reduction by MoFe protein in the presence of CO may reflect an altered coupling of proton and electron transfer under the mediated electrochemical control. In future work it will be productive to examine the pH dependence of the CO effect on electrocatalytic proton reduction, and to examine the alternative nitrogenases under similar conditions. The effect of CO on the electroenzymatic proton reduction current at −0.9 V indicates that CO binds at a redox level populated during H^+^ reduction. Electroenzymatic reduction of C_2_H_2_ by MoFe protein electrodes requires a similar potential to the onset of H^+^ reduction suggesting that this reaction may also proceed from a common redox level. This would be consistent with the proposal that a single redox couple is involved at the FeMo-cofactor core of nitrogenase, with successive electrons accumulated on coordinated substrates.^1^

Binding of carbon monoxide to the FeMo cofactor of MoFe nitrogenase is confirmed in IR spectroelectrochemical experiments (Fig. 2), in which *v*_CO_ absorption bands consistent with CO coordinated to metal sites appear at −0.9 V, and grow in further at −1.1 V, but are absent in analogous experiments on apo protein. Although the electrochemical experiment in Fig. 1 reveals no recovery of current when CO was flushed out with Ar at −0.9 V, reversibility of CO binding is seen in the IR spectra when the potential is stepped back up to 0 V. The experiment did not include mediators to cover the window around 0 V, but the timeframe for this experiment allows for slower direct electron transfer from the electrode to the MoFe protein without mediators. The CO bands from CO interaction at low potential are broad and difficult to assign to specific binding modes and states, and signal/noise is quite low due to the low coverage of protein on the electrode, but the *v*_CO_ bands are in a similar spectral region to the bands observed in stopped flow studies of nitrogenase under ‘hi-CO’ conditions by George *et al.*^14^ Temporal dependence of the appearance of *v*_CO_ bands at −0.9 V (Fig. 4) suggests separate binding modes, which could relate to the lo- and hi-CO conditions identified in earlier studies.^14–16,23^ We do not try to offer a more complete interpretation of the CO binding sites to the FeMo-cofactor from these spectra, but note that the series of spectra provide clear evidence for the onset of CO binding to the FeMo-cofactor occurring at around −0.9 V, just slightly negative of the onset potential for proton reduction, with CO also inhibiting proton reduction at −0.9 V.

Consistent with the observation that MeNC is reduced by MoFe nitrogenase at higher *E*_*n*_ levels than required for reduction of N_2_, we find evidence for binding of MeNC with no applied potential, and for electroenzymatic reduction of MeNC at a mildly reducing potential, −0.1 V, as evidenced by bands in the IR indicative of methylamine formation and depletion of MeNC. In future studies it will be helpful to include redox mediators to cover the 0 to −0.5 V window to achieve more effective control over the enzyme in this regime.

Further work is needed to assess how the potentials measured in this study relate to the situation during native catalytic N_2_ reduction by the enzyme system with Fe protein/ATP as electron donor, where conformational changes related to association/dissociation of the two proteins may link the delivery of electrons to particular stages of the catalytic cycle. We have not tried to ascribe the potentials to particular *E*_*n*_ levels of the MoFe protein at this stage. Future studies might involve spectroscopic study of electrochemically generated levels of the MoFe protein by electron paramagnetic resonance (EPR) spectroscopy, for example, to establish these links. Nevertheless, we are able to provide clear evidence for electrocatalytic H^+^ and C_2_H_2_ reduction by nitrogenase MoFe protein, and distinct onset potentials for binding of CO and MeNC, as represented in Fig. 9.

In previous work we showed that an electron-transfer variant of the MoFe protein catalyses hydrazine to ammonia conversion when reduced with Eu(ii)-polyaminocarboxylate complexes,^24^ and Fe-protein free reduction of N_3_^−^ and NO_2_^−^ to ammonia with cobaltacene as the reductant has been shown by Milton *et al.* at *ca.* −1 V *vs.* SHE,^9^ in a similar potential range as observed here for the onset of H^+^ or C_2_H_2_ reduction or binding of CO as inhibitor, suggesting common redox potentials required for these transformations in the enzyme. The present study was carried out predominantly under an argon atmosphere to remove the possibility of N_2_ interaction. No evidence for electrocatalytic N_2_ reduction was observed when a potential step sequence was performed under N_2_, but further work should examine the potentials for electrocatalytic reduction of different nitrogen-based substrates, and to try to understand the barriers to electrocatalytic N_2_ reduction by the enzyme. So far, Fe-protein free N_2_ reduction has been observed for MoFe protein immobilised on CdS quantum dots,^25,26^ for which it is difficult to assign the potential of electron delivery.

## Conclusions

We apply a combined electrochemical/IR/GC method to define potential windows for proton reduction, MeNC binding and reduction, C_2_H_2_ reduction and CO binding. The potentials for electroenzymatic proton- or acetylene-reduction and the inhibition by CO are grouped around −0.9 V, suggesting a common redox level required for these events. In contrast, MeNC interacts at a much less negative potential. This approach, and these findings, should prompt further studies to explore in more detail the potential landscape for binding of nitrogenase substrates and inhibitors.

## Author contributions

T. C. carried out the investigation and performed formal analysis of data and interpretation. P. A. A. assisted in experimental set-up and advised on formal analysis of data. L. C. S., P. A. A. and K. A. V. conceptualised the study and assisted in interpretation of data. K. A. V. drafted the manuscript. All authors edited the manuscript.

## Conflicts of interest

There are no conflicts to declare.

## Supplementary Material

FD-243-D2FD00170E-s001
