# Supplementary material for: Electrochemical experiments define potentials associated with binding of substrates and inhibitors to nitrogenase MoFe protein
Source: Faraday Discuss. 2023 Feb 6;243:270–86. doi: 10.1039/d2fd00170e (PMC10354833; doi:10.1039/d2fd00170e)
Supplement: FD-243-D2FD00170E-s001 [file FD-243-D2FD00170E-s001.pdf]

Electronic Supplementary Material (ESI) for Faraday Discussions.
This journal is © The Royal Society of Chemistry 2023

Supplementary Information

Electrochemical experiments define potentials associated with binding of substrates and inhibitors to nitrogenase MoFe protein

Ting Chen,[a,b] Philip A. Ash,[a,c,d] Lance C. Seefeldt,[e] Kylie A. Vincent *[a]

Supplementary Methods: Preparation of redox mediator cocktail

Samples of BAPTA (4.76 mg), EGTA (3.8 mg), DTPA (3.93 mg) (Sigma, see Scheme 1) and Eu_2O_3 (5.28 mg, Alfa Aesar) were weighed and taken into an anaerobic glovebox. The polyaminocarboxylate ligands were each dissolved, separately, in 200 µL of 0.25 M NaOH solution (deoxygenated); Eu_2O_3 was dissolved in 300 µL 0.5 M HCl solution (deoxygenated) with the aid of sonication. The Eu^{3+} solution was then divided in three, and added to the ligand solutions evenly to make Eu-L mediator stock solutions. The 0.25 mM mediator solution used in electrochemical experiments is made from adding an aliquot of each mediator stock solution into the relevant amount of Tris-HCl pH 8.0 buffer. Cyclic voltammograms for the individual redox mediator systems are shown in Figure 1.

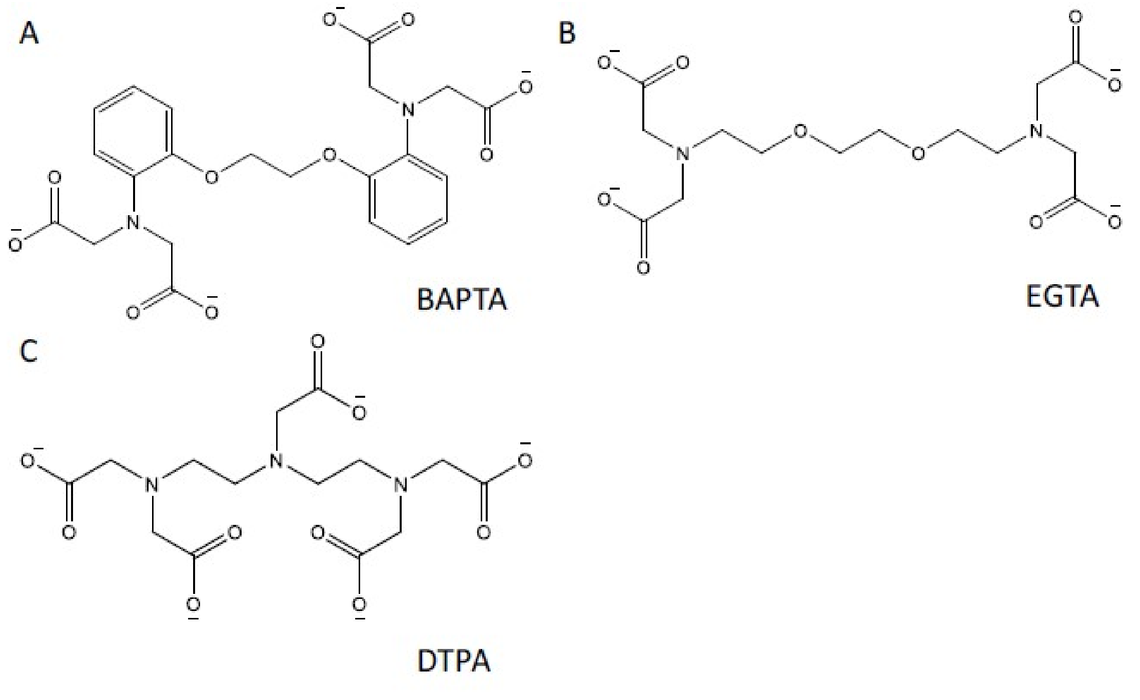

Scheme 1. Ligands used with Eu(III/II) as low potential redox mediators for electrochemically-controlled experiments.

Supplementary experimental data

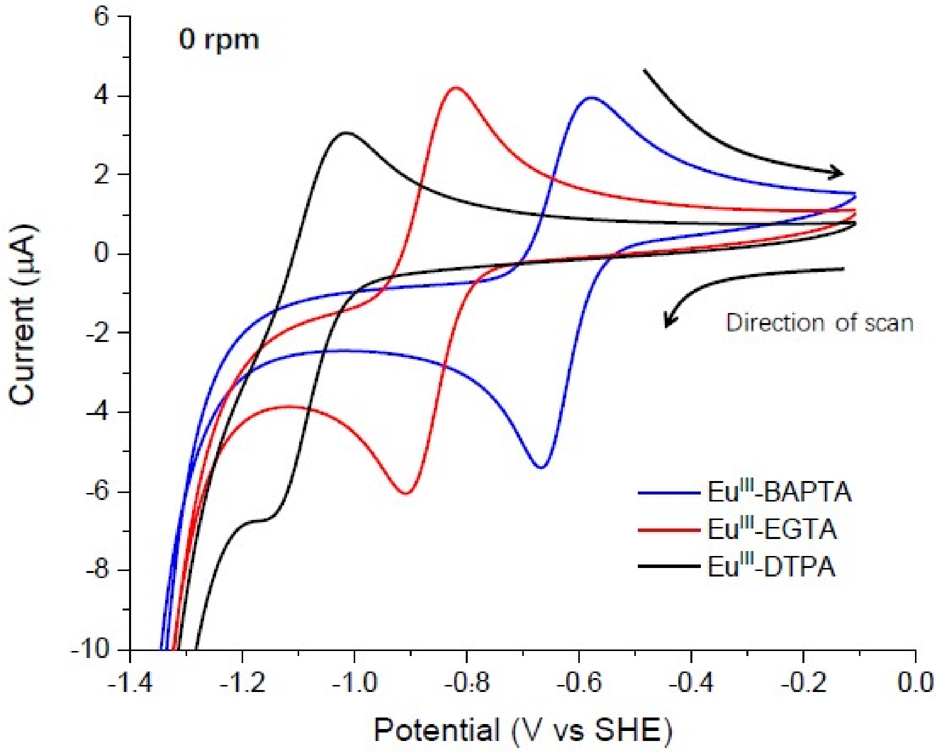

Figure S1. **Cyclic voltammograms of the Eu-L redox mediator components**. Recorded at a stationary electrode, 10 mV s^{-1}.

(A) Direct adsorption

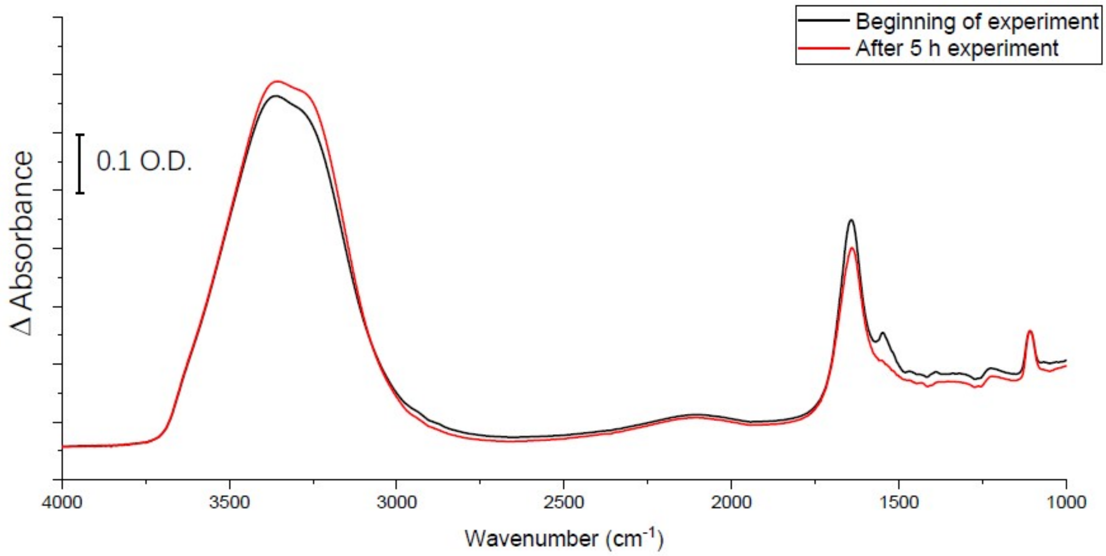

(B) Covalent immobilisation

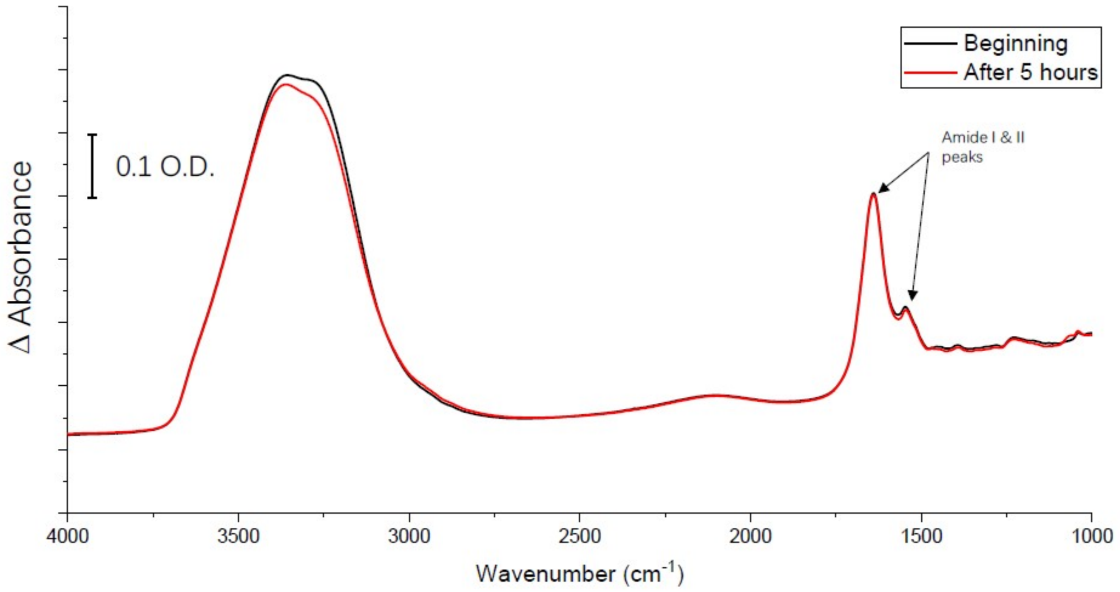

Figure S2. **Immobilisation and stability of nitrogenase MoFe protein on carbon particles.** IR spectra showing nitrogenase native MoFe protein (A) directly adsorbed; and (B) covalently attached to carbon black particles via carbodiimide (EDC-NHSS) coupling, showing the stability of the amide bands over 5 hours for each protein film.

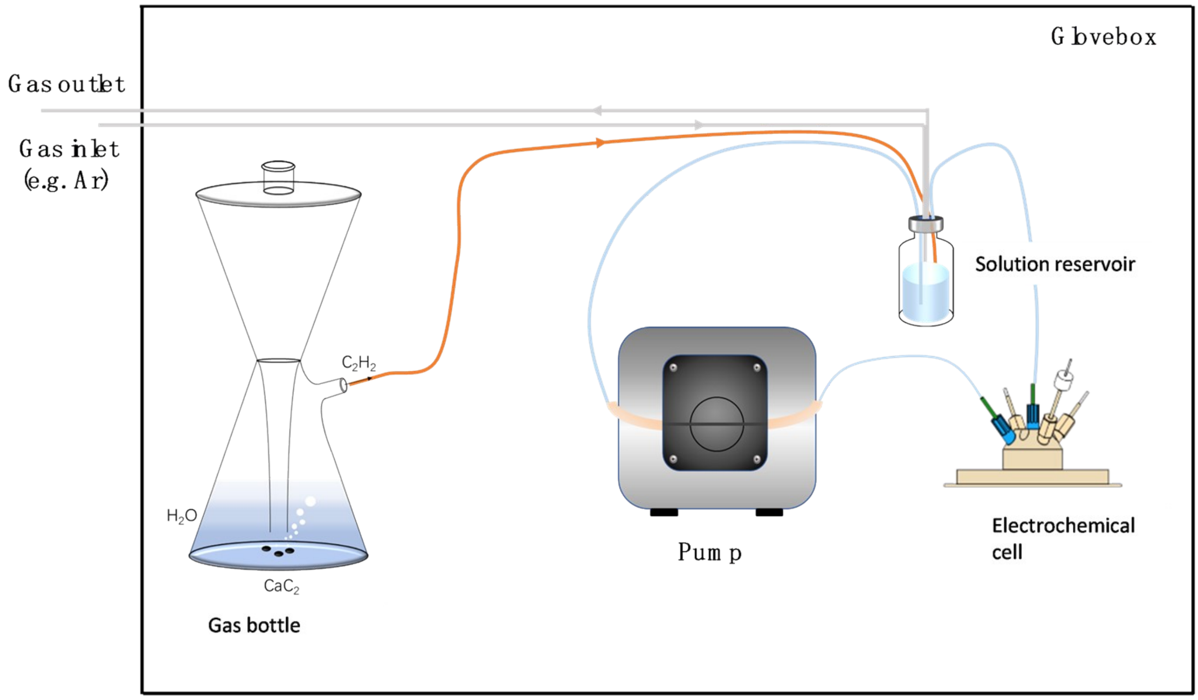

Figure S3. **Schematic diagram of the set-up used for preparation of C_2H_2 from CaC_2.** The C2H2 synthesis in the glass gas bottle flows into the solution reservoir via a syringe through the septum cap. Solution is pumped from the solution reservoir through the electrochemical cell and back to the solution reservoir for further gas equilibration.

(A) CO

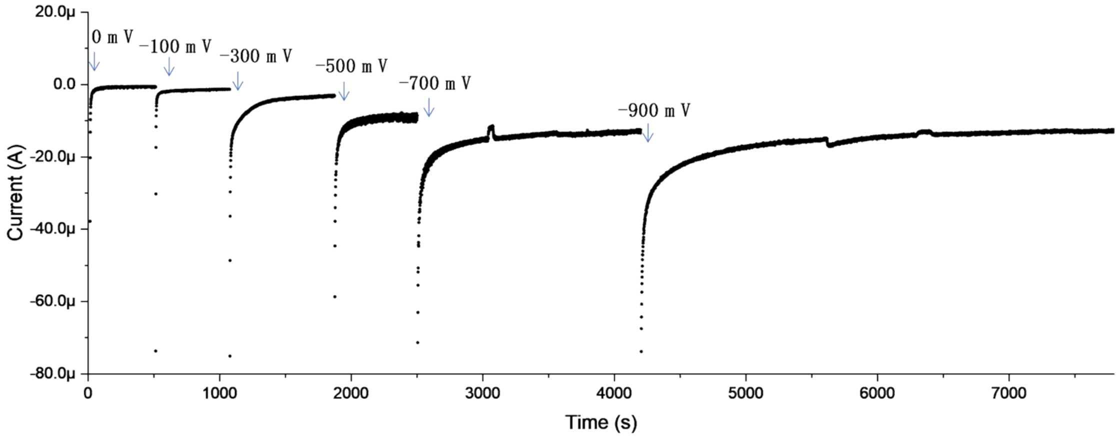

(B) N_2

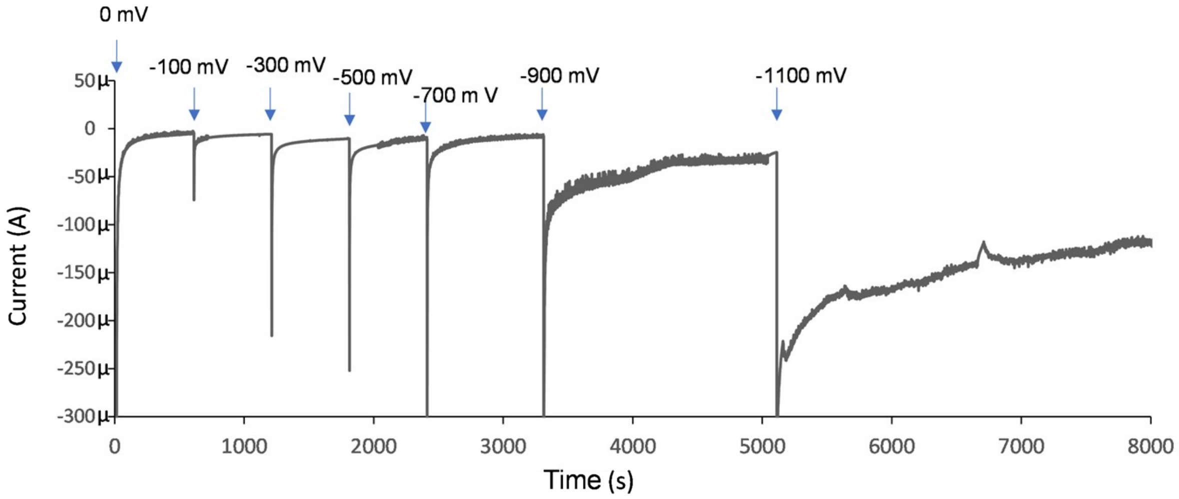

Figure S4. **Potential sequence at a native MoFe protein electrode in the presence of (A) CO and (B) N_2.** Current response for MoFe protein on a carbon black electrode recorded in Tris HCl buffer, pH 8.0, saturated with the gas indicated. Slight 'wobbles' in current (eg at ca 3000 s and 5600 s in panel A) are due to disturbances of the electrochemical cell, for example, when another user put their hands into the glove box gauntlets. The current axes are denoted in Amps (A), and the symbol 'μ' after the numerical values indicates that these are in microAmps, ie number x 10^{-6} A.

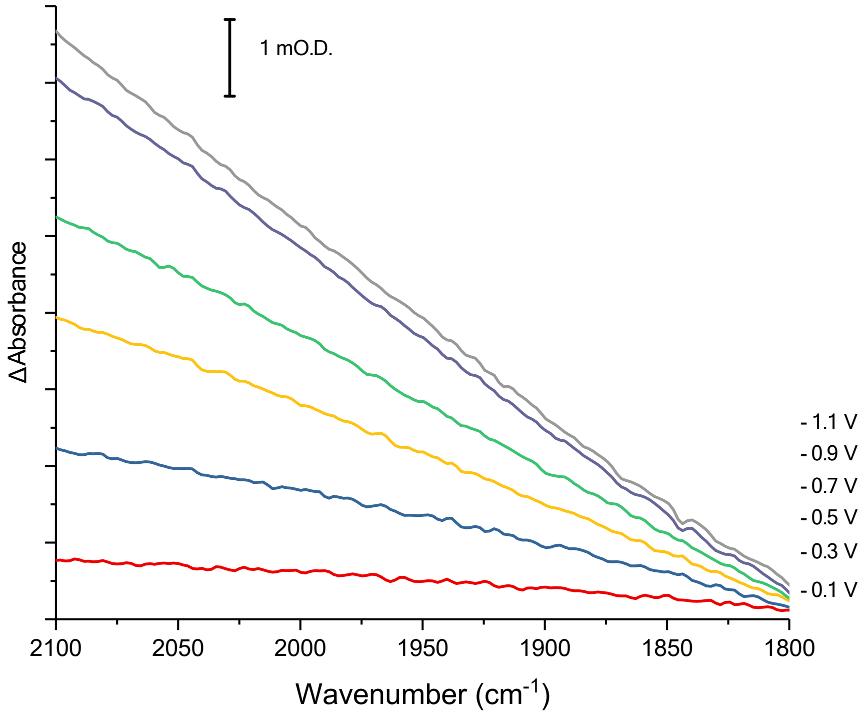

Figure S5. **Control experiment on a bare carbon black electrode in the presence of CO.** Spectral changes observed in the 2100-1800 cm^{-1} region when a carbon-black electrode is stepped from -0.1 to -1.1 mV in 0.2 V intervals. The electrode was unmodified (ie no protein attached); the electrolyte contained Eu-L and was saturated with CO. Spectra are processed against a background recorded at -0.1 V prior to introduction of CO, and were recorded 30 minutes after application of the specified potential.

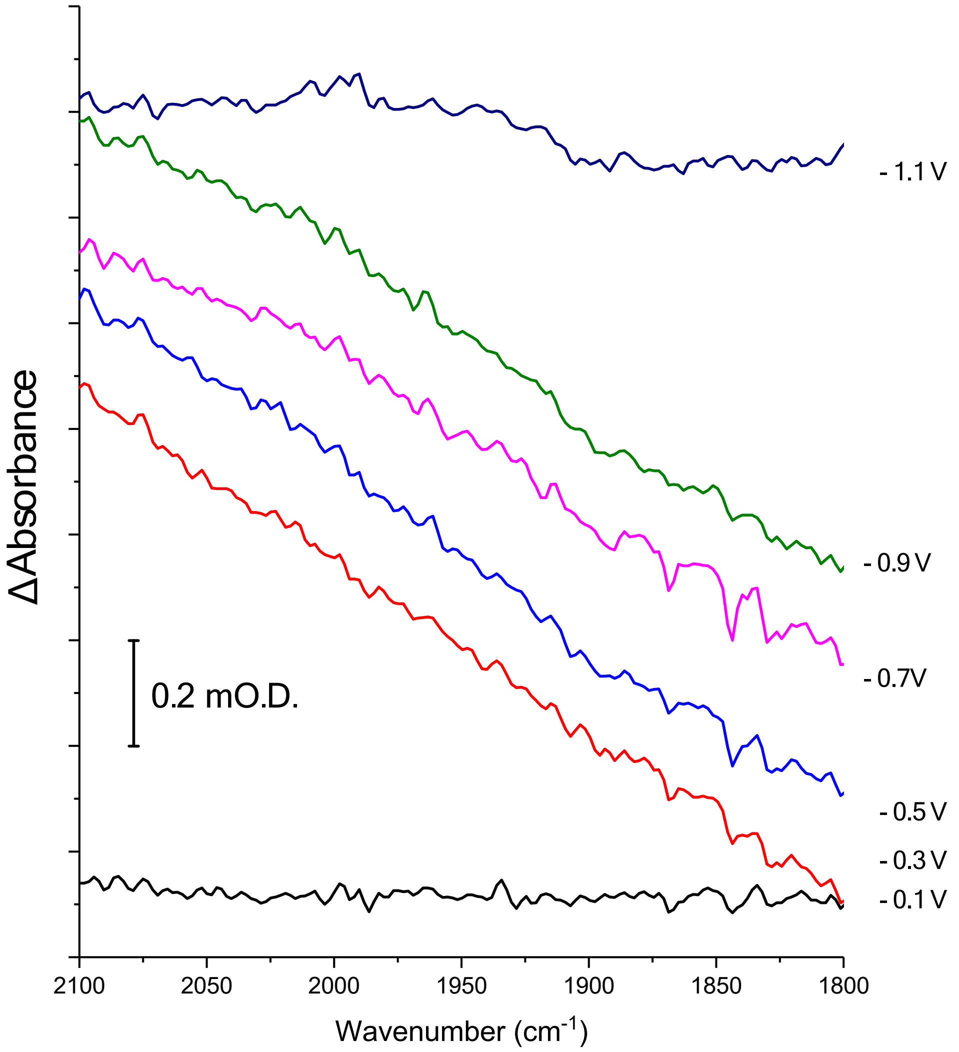

Figure S6. **Control experiment for MoFe protein in the presence of CO in the absence of Eu-L mediator.** Spectral changes observed in the 2100-1800 cm^{-1} region when a carbon-black electrode modified with nitrogenase MoFe protein is stepped from -0.1 to -1.1 mV in 0.2 V intervals in the absence of Eu-L mediator. The electrolyte was Tris HCl pH 8.0 buffer saturated with CO. Spectra are processed against a background recorded at -0.1 V prior to introduction of CO. Spectra for -0.1, -0.3, -0.5 and -0.7 V were recorded 30 minutes after application of the specified potential, while spectra recorded at -0.9 and -1.1 V were recorded 60 minutes after application of the potential.

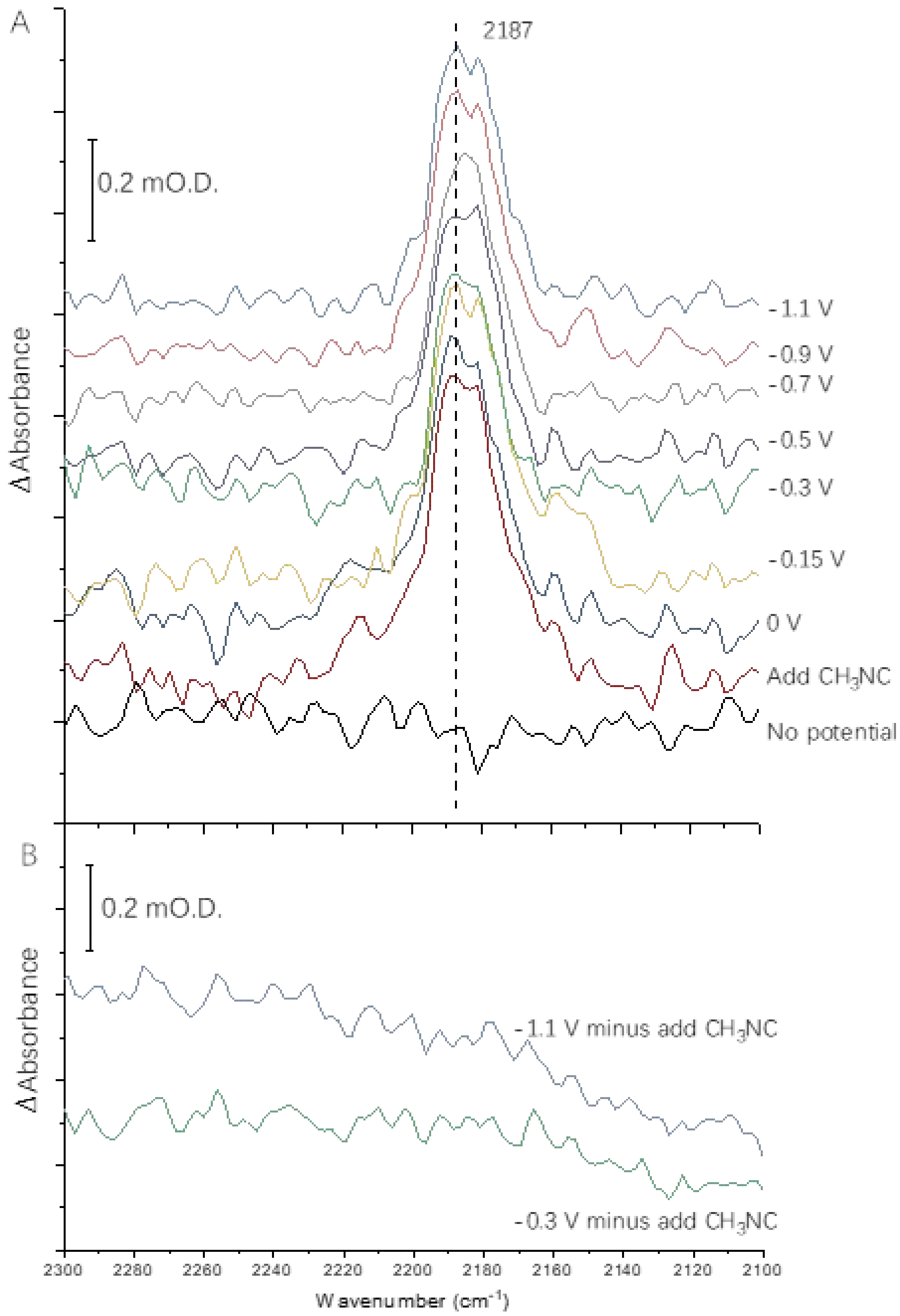

Figure S7. **Control experiment showing no evidence for the MeNC interacting with the apo protein.** IR spectra showing the vNC region recorded for an electrode modified with apo protein on carbon particles in 25 mM methyl isocyanide solution (in Tris-HCl buffer pH 8.0 containing Eu-L mediators) with a series of applied potentials. Each spectrum in (A) was recorded after a 30 min poise at the specified potential and processed against a background spectrum before the addition of methyl isocyanide with no applied potential. The difference spectra in (B) show the change in absorbance at two different potentials relative to a background at 25 mM MeNC with no applied potential.

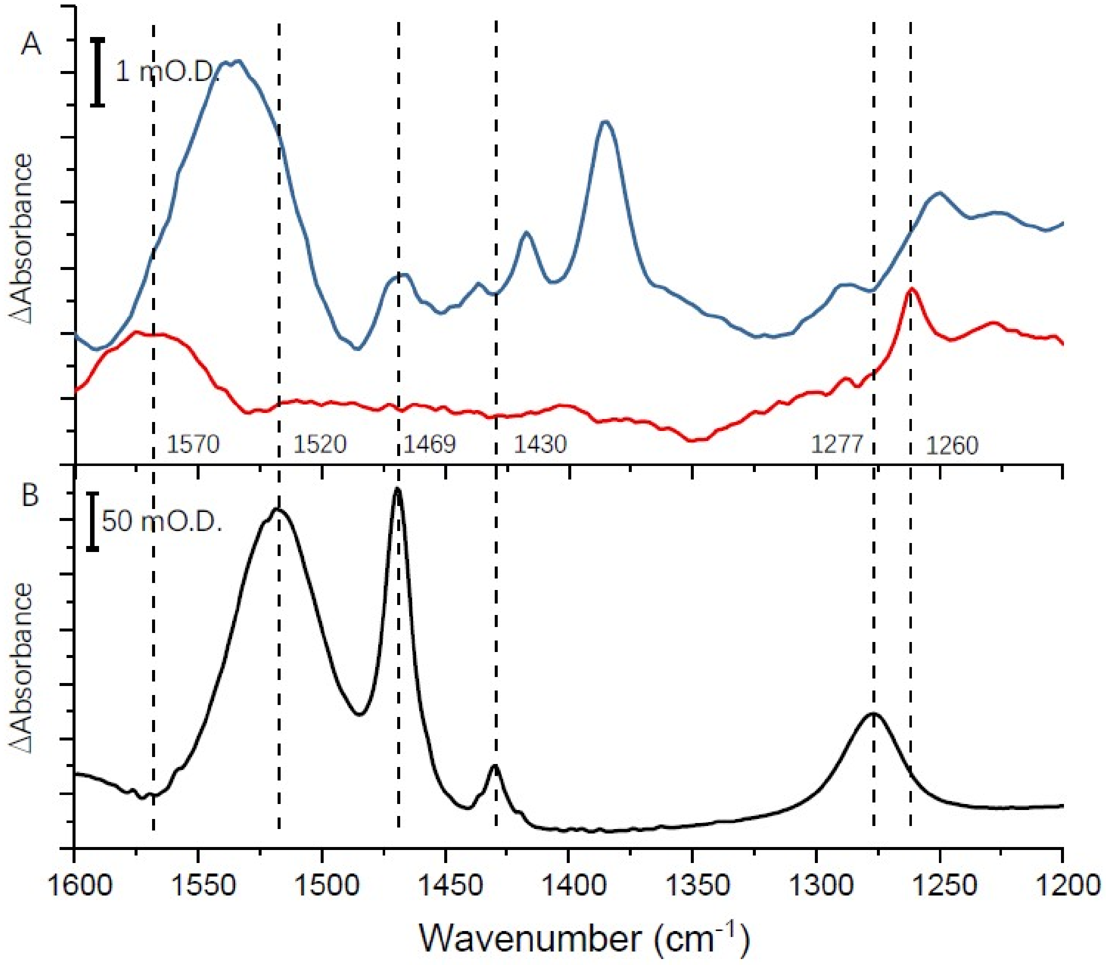

Figure S8. **Control experiment showing that there is no evidence for the formation of methyl amine at an electrode modified with apo protein.** (A) IR spectra for native MoFe protein (blue) and apo protein (red) on a carbon electrode poised at -1.1 V, versus a background spectrum recorded at 0 V with no methyl isocyanide present. These were recorded in Tris HCl pH 8.0 solution containing Eu-L at 25 mM MeNC. (B) Spectrum of 1.5 M methyl amine in Tris HCl pH 8.0 buffer, collected as 1024 co-added scans at 2 cm^{-2} resolution.

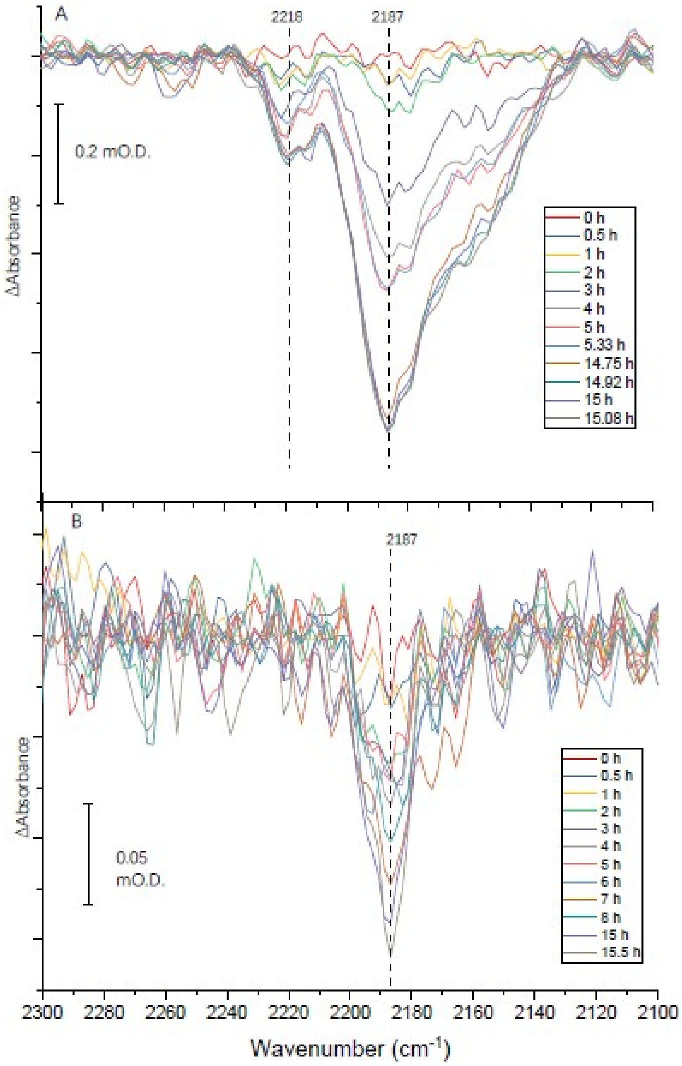

Figure S9. **Evidence for consumption of MeNC for an electrode poised at -1.1 V with either native MoFe protein or apo protein.** Difference spectra recorded over time for an electrode modified with (A) native MoFe protein and (B) apo protein. This experiment was performed with 4 nmol protein immobilised on the electrode, in contrast to other experiments in this study which were performed with 2 nmol protein. The electrode is poised at -1.1 V with the solution stationary in the cell, ie not pumped through. Tris HCl buffer, pH 8.0, with Eu-L and a starting concentration of 25 mM MeNC (starting absorbance 1.19 mO.D. at 2187 cm^{-1}).
